# Time to treatment and mortality for clinical sepsis subtypes

**DOI:** 10.1186/s13054-023-04507-5

**Published:** 2023-06-15

**Authors:** Anne Yang, Jason N. Kennedy, Katherine M. Reitz, Gary Phillips, Kathleen M. Terry, Mitchell M. Levy, Derek C. Angus, Christopher W. Seymour

**Affiliations:** 1 Division of Pulmonary, Allergy, Critical Care, and Sleep Medicine, University of Pittsburgh Medical Center, PA Pittsburgh, USA; 2 Clinical Research, Investigation, and Systems Modeling of Acute Illness (CRISMA) Center, Pittsburgh, PA USA; 3 Department of Critical Care Medicine, University of Pittsburgh Medical Center, Pittsburgh, PA USA; 4 Department of Surgery, Division of Vascular Surgery, University of Pittsburgh Medical Center, Pittsburgh, PA USA; 5 The Ohio State University, Center for Biostatistics, Columbus, OH USA; 6 Lightning Strategies LLC, New York City, NY USA; 7 Division of Pulmonary, Critical Care and Sleep Medicine, Warren Alpert Medical School at Brown University, Providence, RI USA; 8 Department of Emergency Medicine, University of Pittsburgh Medical Center, Pittsburgh, PA USA

**Keywords:** Sepsis, Subtypes, Precision medicine, Antibiotics

## Abstract

**Background:**

Sepsis is common, deadly, and heterogenous. Prior analyses of patients with sepsis and septic shock in New York State showed a risk-adjusted association between more rapid antibiotic administration and bundled care completion, but not an intravenous fluid bolus, with reduced in-hospital mortality. However, it is unknown if clinically identifiable sepsis subtypes modify these associations.

**Methods:**

Secondary analysis of patients with sepsis and septic shock enrolled in the New York State Department of Health cohort from January 1, 2015 to December 31, 2016. Patients were classified as clinical sepsis subtypes (α, β, γ, δ-types) using the Sepsis ENdotyping in Emergency CAre (SENECA) approach. Exposure variables included time to 3-h sepsis bundle completion, antibiotic administration, and intravenous fluid bolus completion. Then logistic regression models evaluated the interaction between exposures, clinical sepsis subtypes, and in-hospital mortality.

**Results:**

55,169 hospitalizations from 155 hospitals were included (34% α, 30% β, 19% γ, 17% δ). The α-subtype had the lowest (N = 1,905, 10%) and δ-subtype had the highest (N = 3,776, 41%) in-hospital mortality. Each hour to completion of the 3-h bundle (aOR, 1.04 [95%CI, 1.02–1.05]) and antibiotic initiation (aOR, 1.03 [95%CI, 1.02–1.04]) was associated with increased risk-adjusted in-hospital mortality. The association differed across subtypes (p-interactions < 0.05). For example, the outcome association for the time to completion of the 3-h bundle was greater in the δ-subtype (aOR, 1.07 [95%CI, 1.05–1.10]) compared to α-subtype (aOR, 1.02 [95%CI, 0.99–1.04]). Time to intravenous fluid bolus completion was not associated with risk-adjusted in-hospital mortality (aOR, 0.99 [95%CI, 0.97–1.01]) and did not differ among subtypes (p-interaction = 0.41).

**Conclusion:**

Timely completion of a 3-h sepsis bundle and antibiotic initiation was associated with reduced risk-adjusted in-hospital mortality, an association modified by clinically identifiable sepsis subtype.

**Supplementary Information:**

The online version contains supplementary material available at 10.1186/s13054-023-04507-5.

## Introduction

Sepsis is a dysregulated response to infection that leads to life-threatening organ dysfunction, accounting for millions of global deaths annually [1, 2]. The host, pathogen, and host response to infection all contribute to broad syndrome heterogeneity, challenging the development of precision treatment [3].

The mainstays of current international clinical guidelines include prompt sepsis identification, measurement of serum lactate, collection of peripheral blood cultures, administration of appropriate antibiotics, and fluid resuscitation with ongoing hemodynamic support [4]. Broadly, this care bundle may improve sepsis-related outcomes, but there is debate about its use [5, 6]. To better identify and quantify sepsis heterogeneity, recent work describes various clinical subtypes, identified using multi-omic profiles, clinical data, or knowledge-based classification [7–9]. To date, little work evaluates the association of bundled care and outcome across different domains of sepsis heterogeneity.

To address the knowledge gap, we used data from New York State Department of Health (NYSDOH) clinical sepsis database to test whether clinically identifiable sepsis subtypes modify the risk-adjusted association between prompt sepsis treatment and outcome during statewide mandated sepsis emergency care.

## Methods

We conducted a secondary analysis of patients with community-acquired sepsis and septic shock as reported to the NYSDOH from 192 hospitals (January 1, 2015 to December 31, 2016). This study was approved with a waiver of informed consent by the NYSDOH institutional review boards and follows the Strengthening the Reporting of Observational Studies in Epidemiology (STROBE) guidelines [10].

### Sepsis protocols and bundles

In 2013, the NYSDOH began a statewide mandate for sepsis protocols facilitating the early identification and treatment of severe sepsis or septic shock (NY Codes, Rules and Regulations 405.4) [11]. Prior work analyzed patients enrolled from April 1, 2014 to June 30, 2016 [12]. Now, the current work includes patients enrolled through December 31, 2016 with updated follow-up.

Sepsis protocols mandated 3- and 6-h bundles of care for all patients diagnosed with community-acquired sepsis, as defined by Sepsis-2 (eMethods) [12, 13]. The 3-h bundle included three items, 1) serum lactate measurement, 2) blood culture collection prior to antibiotic administration, and 3) receipt of broad-spectrum antibiotics. Time from protocol initiation to bundle completion was documented for the subsequent 12 h. The 6-h bundle required consideration of an additional three items, 1) completion of an initial 30 mL per kg of body weight intravenous fluid bolus in patients with hypotension or serum lactate elevation (≥ 4.0 mmol/L), 2) initiation of vasopressors for hypotension refractory to intravenous fluids, and 3) repeated serum lactate measurement, if initial lactate ≥ 4.0 mmol/L. Time from protocol initiation to intravenous bolus completion was documented for the subsequent 6 h. The NYSDOH mandated care items from each bundle to be initiated within 3- or 6-h of Emergency Department (ED) presentation, as appropriate.

### Data sources

Hospital characteristics were abstracted from the NYSDOH administrative database and were linked to validated electronic hospitalization-report forms mandated by the NYSDOH sepsis protocols for patient level data [12]. Data forms included demographics and comorbid conditions defined by the Elixhauser Comorbidity Index, time-date stamped physiologic and laboratory characteristics, as well as in-hospital outcomes. Missing data were quantified using multiple imputation by chained equations (eMethods).

### Sepsis cohort

We included hospitals with NYSDOH sepsis protocols with both 3- and 6-h bundles and excluded hospitals with fewer than 50 annual hospitalizations for sepsis. To study community-acquired sepsis, we included hospitalizations with Sepsis-2 defined severe sepsis or septic shock [12, 13]. The internal validity of Sepsis-2 reporting is described elsewhere [12]. We excluded hospitalizations for patients younger than 18 years of age, with clinical contraindications or advanced directive present, those who declined participation in sepsis bundles, and those transferred into or out from the hospital of record (eMethods). We also excluded hospitalizations when sepsis protocol was initiated outside of the Emergency Department, such as pre-hospital, or greater than six hours after ED arrival. These cases are outside the window of mandated care or may not represent community-acquired sepsis. Though the mandate for 30 mL per kg applies specifically to sepsis patients with hypotension or a serum lactate level ≥ 4.0 mmol/L, we analyzed all sepsis patients who completed the intravenous fluid bolus within six hours. We excluded hospitalizations for sepsis patients when the weight-based intravenous fluid bolus was initiated but not completed within six hours.

### Variables

The primary outcome was in-hospital mortality. Secondary outcomes were Intensive Care Unit (ICU) admission and hospital length of stay (days). Exposures were quantified from time of sepsis protocol initiation to the time of bundle or bundle item completion, as recorded in the NYDOH clinical database. We evaluated three exposures as continuous variables, the time (in hours) to, (i.) 3-h bundle completion, (ii.) antibiotic initiation, and among those meeting criteria for treatment, (iii.) initial weight-based intravenous fluid bolus completion. For the 3-h bundle and antibiotic intervention, time to initiation was documented from protocol initiation through the subsequent 12 h. The time to completion of an initial intravenous fluid bolus was documented from protocol initiation through six hours. To determine clinical sepsis subtypes, we used the Sepsis ENdotyping in Emergency CAre (SENECA) criteria [7]. Of the 29 variables proposed in SENECA subtype methods, we used all variables available in the NYSDOH dataset. These included continuous (i.e., age and serum lactate level [mmol/L]) and dichotomous variables (i.e., sex, bandemia [> 5% band count], presence of comorbid conditions, altered mental status, and thrombocytopenia [< 150,000 cells/mm^3^]). The Euclidean distance was calculated to the published SENECA derivation subtype centroid, after which the minimal distance identified the patient as α, β, γ, or δ sepsis subtype [11].

### Statistical analysis

We compared hospitalization data across the clinical sepsis subtypes (α, β, γ, δ). Continuous data were expressed as means with standard deviations or as medians with interquartile ranges (IQR). Categorical variables are shown as frequency and percentages. Descriptive data were compared using T-test, K-Wallis, and Chi-squared testing, as appropriate. The range and variability in the times to treatments were displayed with cumulative proportions.

We used random-effect logistic regression with hospital as a random variance effect to evaluate the association between in-hospital mortality and time to bundle or bundle element completion. Moderation of the treatment effect by clinical sepsis subtypes was assessed with interaction terms. Models generated adjusted odds ratios (aOR) with 95% confidence intervals (95%CI). Time to bundle or bundle element completion, overall and interacted with subtype, was included as a linear covariate after assessment for nonlinear relationships with the use of fractional polynomials (p > 0.05) for all models (Additional file 1: Fig. S1) [7]. For each clinical sepsis subtype, the adjusted risk of in-hospital mortality across the range of time to bundle or bundle items was estimated using the empirical Bayesian method [7].

All analyses were performed with Stata, v14.2 (StataCorp), PRISM 9.4.1 (GraphPad Software), and OmniGraffle 7.19.4.

### Sensitivity analysis

We completed two sensitivity analyses. First, we computed the E-value to understand the magnitude of a potential unmeasured confounder required to negate the association between time to completion of elements and mortality overall, within each clinical sepsis subtype, and for overall effect modification by subtype [14, 15]. Second, we evaluated the association between the time to other bundle elements including time to (i.) peripheral blood culture collection and (ii) serum lactate measure and their effect modification (or not) by clinical sepsis subtype.

## Results

**Fig. 1:**
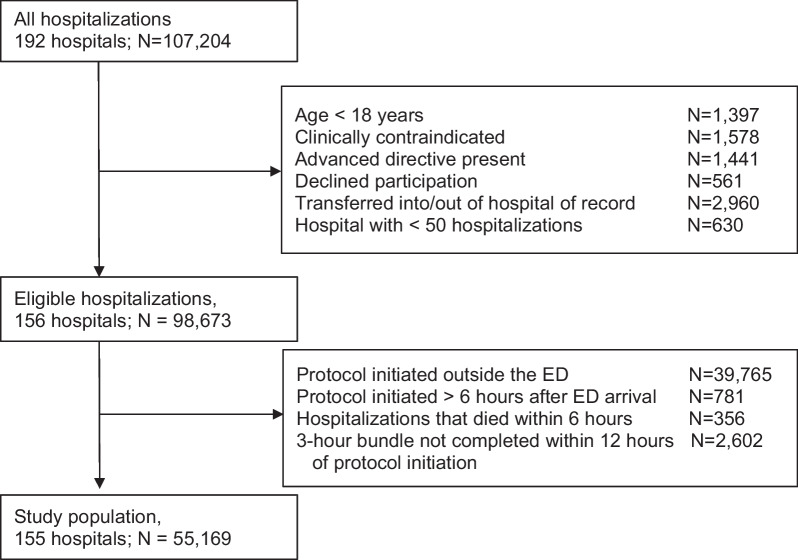
Cohort accrual

Among 107,240 hospitalizations at 192 hospitals, we included 55,169 (51%) adult hospitalizations (median age 72 [IQR, 60–83] years; 52% male: 66% White) with community-acquired sepsis at 155 hospitals (Fig. 1). When classified by clinical sepsis subtype, α-subtype was most common (N = 18,880, 34%), followed by β-subtype (N = 16,381, 30%), γ-subtype (N = 10,704, 19%), and δ-subtype (N = 9,204, 17%). When comparing demographics, coexisting conditions, and sepsis characteristics (Table 1), the β-subtype was older with more comorbid conditions and were more frequently admitted from a nursing facility, when compared to the α-subtype. The δ-subtype presented with the greatest serum lactate concentration (median 7.0 [IQR, 5.2 – 9.6] mmol/L) and highest proportion of septic shock (N = 6,326, 69%), and were the most likely to be admitted to the ICU (N = 6,999, 76%). Among the 55,169 hospitalizations, 26,766 completed an initial intravenous fluid bolus within 6-h of protocol initiation. These hospitalizations had similar distribution by clinical sepsis subtype and characteristics to the full cohort (Additional file 1: Table S1).

**Table 1:** Baseline characteristics

	All	Clinical Sepsis Subtype			
		⍺	β	γ	δ
No. (%)	55,169	18,880 (34)	16,381 (30)	10,704 (19)	9,204 (17)
Patient demographics					
Age at admission, med. [IQR]	72 [60–83]	63 [51–75]	80 [71–87]	73 [62–84]	72 [61–83]
Sex, No. (%)					
Male	28,636 (52)	10,549 (56)	7,847 (48)	4,678 (44)	5,382 (60)
Female	26,533 (48)	8,331 (44)	8,534 (52)	6,026 (56)	3,642 (40)
Race, No. (%)					
White	36,634 (66)	12,019 (64)	11,451 (70)	7,401 (69)	5,763 (63)
Black	9,308 (17)	3,458 (18)	2,554 (16)	1,533 (14)	1,763 (19)
Asian	2,461 (4.5)	768 (4.1)	684 (4.2)	537 (5.0)	472 (5.1)
Other^a^	6,766 (12)	2,635 (14)	1,692 (10)	1,233 (12)	1,206 (13)
Admission source, No. (%)					
Home	38,128 (69)	14,069 (75)	10,187 (62)	7,628 (71)	6,244 (68)
Skilled nursing facility	14,458 (26)	3,867 (21)	5,451 (33)	2,526 (24)	2,614 (28)
Other^b^	2,583 (4.7)	944 (5.0)	743 (4.5)	550 (5.1)	346 (3.8)
Coexisting condition, No. (%)					
Chronic respiratory failure	5,704 (10)	795 (4.2)	3,195 (20)	687 (6.4)	1,027 (11)
Congestive heart failure	10,968 (20)	1,510 (8.0)	6,452 (39)	1,449 (14)	1,557 (17)
End-stage renal disease	5,426 (9.8)	880 (4.7)	3,043 (19)	603 (5.6)	900 (9.8)
Sepsis characteristics					
Site of infection, No. (%)					
Urinary	15,134 (27)	5,719 (30)	4,153 (25)	3,064 (29)	2,198 (24)
Respiratory	22,048 (40)	6,826 (36)	7,628 (47)	3,991 (37)	3,603 (39)
Gastrointestinal	5,586 (10)	1,504 (8.0)	1,422 (8.7)	1,411 (13)	1,249 (14)
Other^c^	12,401 (23)	4,831 (26)	3,178 (19)	2,238 (21)	2,154 (23)
Positive blood cultures, No. (%)	8,111 (15)	2,264 (12)	1,881 (12)	1,977 (19)	1,989 (22)
Lactate^d^, mmol/L, med. [IQR]	2.7 [1.8–4.2]	2.2 [1.5–3.1]	2.3 [1.5–3.3]	2.9 [2.0–4.2]	7.0 [5.2–9.6]
Septic shock^e^, No. (%)	24,182 (44)	4,188 (22)	9,353 (57)	4,315 (40)	6,326 (69)
Hypotension status^f^, No. (%)					
Fluid unresponsive	16,117 (29)	3,126 (17)	5,730 (35)	2,932 (27)	4,329 (47)
Responsive to fluids	16,437 (30)	5,172 (27)	4,376 (26)	3,580 (33)	3,309 (36)
In-hospital Outcomes					
ICU admission, No. (%)	30,923 (56)	7,059 (37)	11,419 (70)	5,446 (51)	6,999 (76)
Length of stay, days, med. [IQR]	8 [5–14]	7 [4–13]	9 [6–16]	8 [5–14]	8 [3–15]
Mortality, No. (%)	11,998 (22)	1,905 (10)	4,450 (27)	1,867 (17)	3,776 (41)

*ICU* intensive care unit, *IQR* interquartile range, *med* median, *LOS* length of stay

^a^Other race corresponds to Chinese, Filipino, Hawaiian, American Indian/Alaskan, Asian, Hawaiian/Other Pacific Islander, Middle Eastern, Native American, or Pacific Islander

^b^Other locations include clinic or unknown

^c^Other sources include skin, central nervous system, and unknown

^d^Corresponds to minimum or maximum values within 6 h of hospital admission

^e^As defined by Sepsis-2 guidelines

^f^Fluid unresponsive defined as hypotension or elevated lactate not responsive to fluids; Responsive to fluids defined as hypotension or elevated lactate responsive to fluid

### Time to treatment by clinical sepsis subtype

**Fig. 2:**
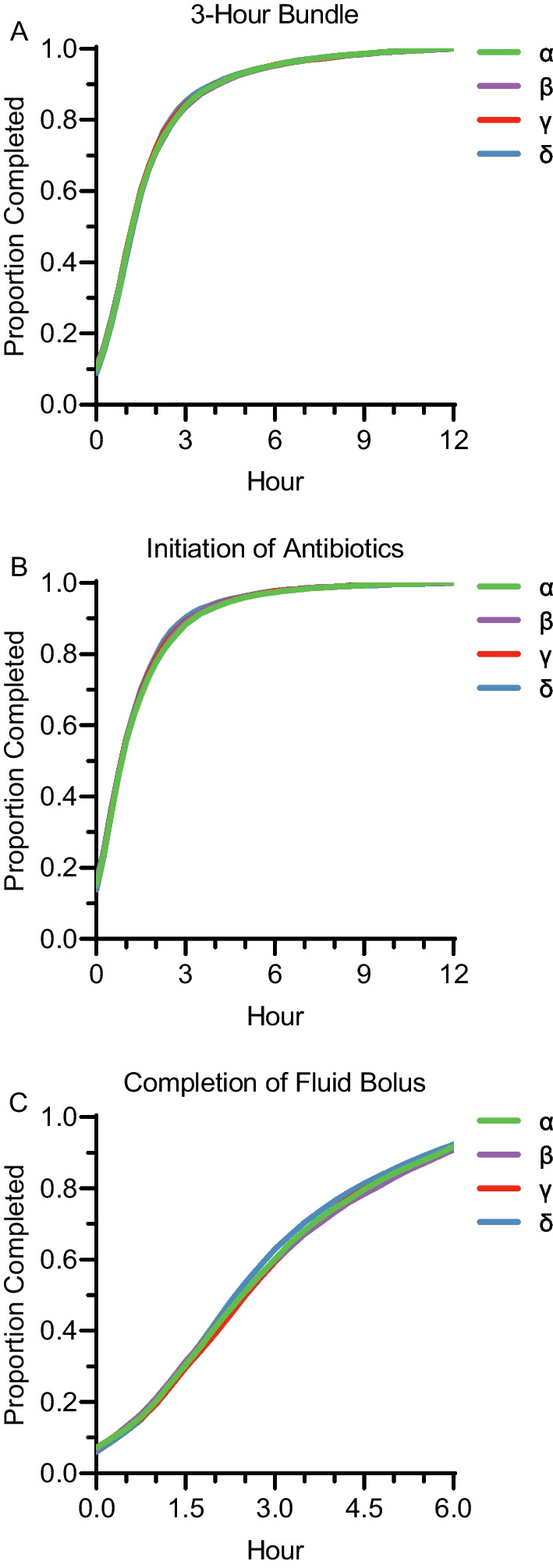
Cumulative proportions of bundle and bundle item completion, stratified by clinical sepsis subtype. Shown are the proportions of cohort completing the 3-h bundle of sepsis care over the first 12-h after protocol initiation (Panel **A**), the administration of broad-spectrum antibiotics over the first 12-h after protocol initiation (Panel **B**), and initial intravenous fluid bolus completion over the first 6-h after protocol initiation (Panel **C**), stratified by clinical sepsis subtype

After sepsis protocol initiation and among patients who completed each respective intervention within 12 h, the median time to the completion of the 3-h bundle and initiation of antibiotic therapy was 1.2 (IQR, 0.6–2.2) and 0.9 (IQR, 0.3–1.8), respectively. Among patients who completed the intravenous fluid bolus within six hours, the median time to completion was 2.5 h (IQR, 1.3–4.1). Time to completion of the 3-h bundle did not differ across sepsis subtypes (α: 1.2 [IQR, 0.6–2.2], β: 1.2 [IQR, 0.5–2.3], γ: 1.2 [IQR, 0.6–2.2], δ: 1.3 [IQR, 0.6–2.1] hours, p = 0.36). Similarly, the time to antibiotic initiation did not differ across sepsis subtypes (α: 0.9 [IQR, 0.3–1.9], β: 0.9 [IQR, 0.3–1.8], γ: 0.9 [IQR, 0.3–1.8], δ: 0.8 [IQR, 0.2–1.7] hours, p = 0.079). The time to initial intravenous fluid bolus completion did statistically, but not clinically, significantly differ across sepsis subtypes (α: 2.5 [IQR, 1.3–4.1], β: 2.5 [IQR, 1.2–4.2], γ: 2.5 [IQR, 1.3–4.1], δ: 2.4 [IQR, 1.3–3.9] hour, p = 0.013; Fig. 2, Additional file 1: Table S6).

### Primary analysis

One in five sepsis hospitalizations did not survive to discharge (N = 11,998, 22%). In-hospital mortality was highest in the δ-subtype (N = 3,776, 41%), compared to β-subtype (N = 4,450, 27%), γ-subtype (N = 1,867, 17%), and α-subtype (N = 1,905, 10%; Table 1). After regression modeling, each hour of time to both the completion of the 3-h bundle (aOR, 1.04 [95%CI, 1.02–1.05]) and initiation of antibiotics (aOR, 1.03 [95%CI, 1.02–1.04]) was associated with an increased risk of in-hospital mortality. However, each hour of time to intravenous fluid bolus completion was not associated with the risk of in-hospital mortality (aOR, 0.99 [95%CI, 0.97–1.01]).

**Fig. 3:**
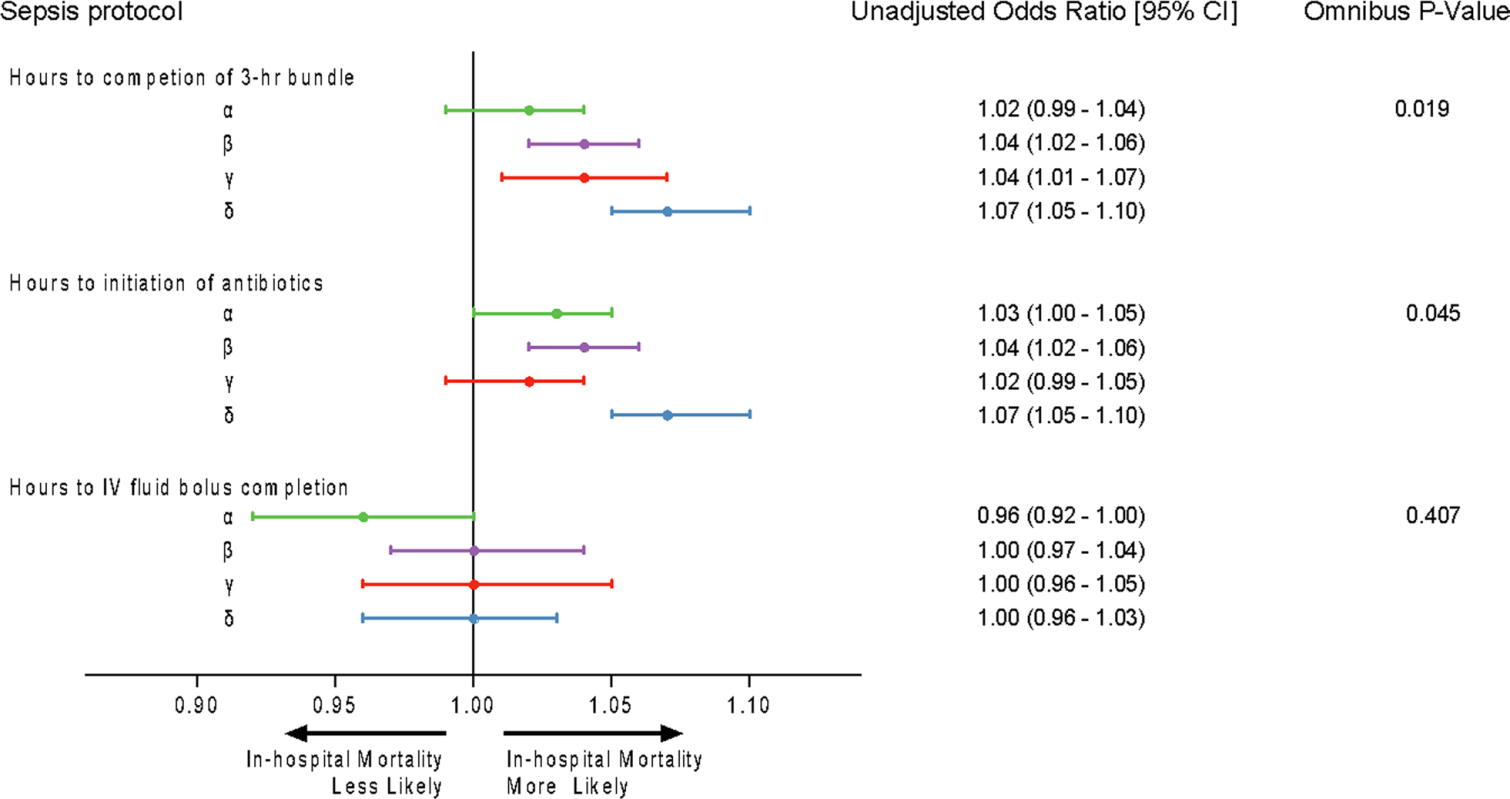
Adjusted odds ratios of in-hospital mortality, stratified by clinical sepsis subtype. Shown are odd ratios with 95% confidence intervals for in-hospital mortality, per hour, to completion of the 3-h bundle, initiation of antibiotics, and completion of the initial intravenous fluid bolus completion, by clinical sepsis subtype. ^a^Omnibus P values for interaction between sepsis protocol items as well as the sepsis subtypes

**Fig. 4:**
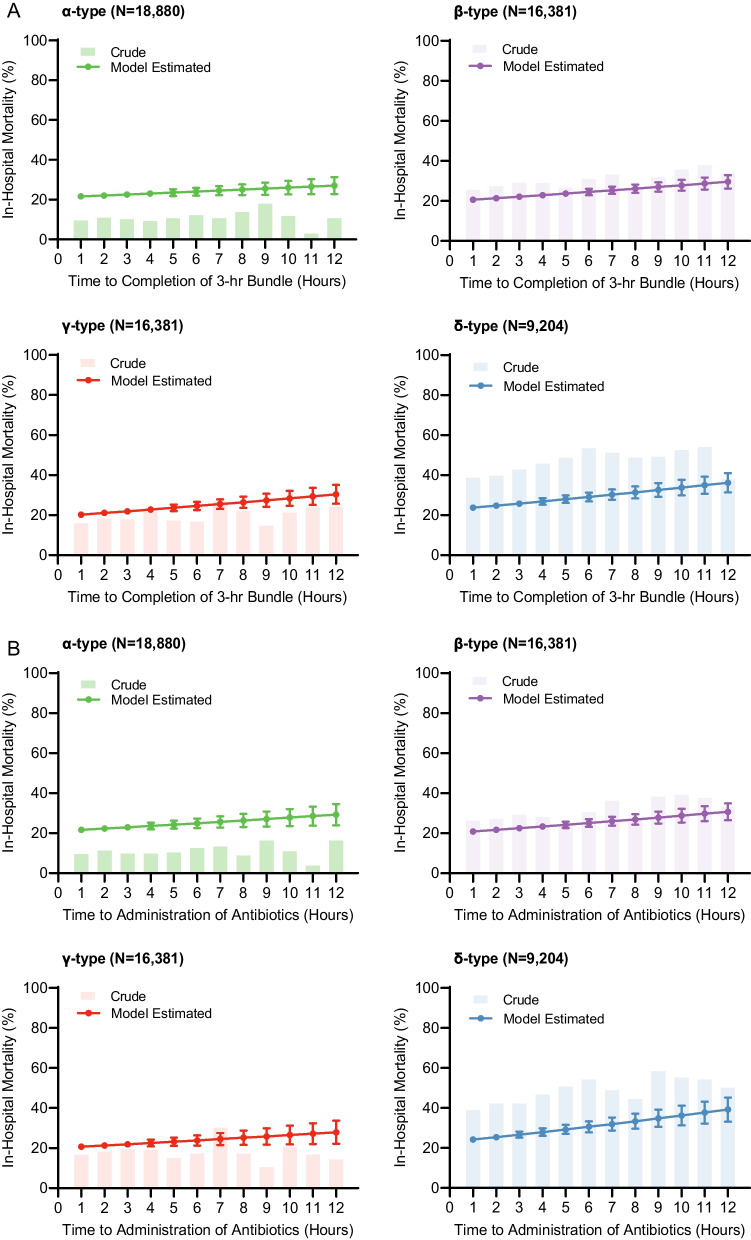
Crude in-hospital mortality and predicted risks of in-hospital death by clinical sepsis subtype. Shown are the crude in-hospital mortality and model-estimated risks of in-hospital mortality for each clinical sepsis subtype, across a range of times from initiation of protocol to completion of the 3-h bundle (Panel **A**) and initiation of antibiotics (Panel **B**). Bars associated with model-estimated risks represent 95% confidence

The association between time to treatment and in-hospital mortality significantly differed among clinical sepsis subtypes for time to completion of the 3-h bundle and initiation of antibiotics (p-interaction < 0.05; Figs. 3, 4, Additional file 1: Table S2, S3, S7). This was not the case for intravenous fluid bolus completion (p-interaction = 0.41; Additional file 1: Table S4, Additional file 1: Fig. S2). For example, the association between each hour of time to completion of the 3-h bundle and increased in-hospital mortality was greatest in the δ-subtype (aOR, 1.07 [95%CI, 1.05–1.10]) as compared to the α-subtype (aOR, 1.02 [95%CI, 0.99–1.04]).

When comparing the completion of the 3-h bundle at six hours to one hour after sepsis protocol initiation, there was a 5% increase in the adjusted risk of in-hospital mortality for δ-subtype (completion at six hours, 29% [95% CI: 27%-31%] vs one hour, 24% [95%CI, 23%-25%] mortality) and a 2% increase for α-subtype (completion at six hours, 24% [95%CI, 22%-26%] vs one hour 22% [95%CI, 20%-23%] mortality; Fig. 4). The random effect logistic regressions presented in this work provide estimates of the relationship between delay in treatment elements and mortality, modified by clinical sepsis subtype. In Fig. 4, we showed the marginal probability of in-hospital mortality and crude mortality for each clinical sepsis subtype, aggregated hourly.

### Sensitivity analysis

The association between time to completion of the 3-h bundle and initiation of antibiotics would require an unmeasured confounder with an aOR of at least 1.16 (lower limit CI, 1.11) and 1.14 (lower limit CI, 1.11) to negate the primary analysis findings. Among clinical sepsis subtypes, the δ-subtype E-value was larger in magnitude (3-h bundle, 1.22 [lower limit CI, 1.18] and initiation of antibiotics, 1.22 [lower limit CI: 1.18]) than other sepsis subtypes; Additional file 1: Tables S6–S8). Notably, the association between time to obtaining peripheral blood cultures and initial lactate measurement was not significantly different among clinical sepsis subtypes (blood culture p-interaction = 0.064; lactate p-interaction = 0.52) (Additional file 1: Table S5).

## Discussion

In a statewide cohort of mandated protocolized sepsis care in the emergency department, we observed an association between time to treatment and outcome, a finding modified by clinical sepsis subtype. Specifically, the association between time to completion of the 3-h bundle and administration of antibiotics with risk-adjusted in-hospital mortality was greatest among the δ-subtype and lowest among the α-subtype. We did not find effect modification for time to completion of the initial intravenous fluid bolus and in-hospital mortality by clinical sepsis subtype.

For over two decades, the association between timely sepsis care and improved outcomes has been well-described. Prior work in a similar statewide cohort from the NYSDOH demonstrated a risk-adjusted association between both more rapid treatment with antibiotics and more rapid completion of the 3-h bundle with reduced in-hospital mortality for sepsis patients [12]. Our work extends these findings and explores effect modification of these elements by clinical sepsis subtype derived using machine learning. Many call for a precision approach, and the National Institutes of Health Working Group on Sepsis recommended greater emphasis on unpacking the complexity and heterogeneity of sepsis [5, 20]. To this end, we respond and extend prior findings by linking the largest statewide database of mandated sepsis care to machine-learned, clinically computable, biologically relevant sepsis subtyping.

There are many potential mechanisms for a differential association between timely sepsis treatment in the ED and outcomes across clinical sepsis subtypes. First, the conceptual rationale has face validity, which advances that one sepsis patient does not always need to or respond to the same treatment as the second [21]. This is observed in studies of surgical source control, for example, where some surgical interventions were associated with improved risk-adjusted outcomes if performed within 12 h of sepsis onset, while others were not [22]. Second, clinical sepsis subtypes are not just markers of severity. Prior work demonstrated effect modification for time to sepsis treatment, particularly in older adults and those with greater illness severity. This work explored high risk subgroups compared to those younger and less critically ill [17–19]. Our work extends that finding by further demonstrating effect modification by clinical sepsis subtype, machine-learned using unsupervised models in external datasets.

Clinical sepsis subtypes likely capture different underlying biologic mechanisms than just levels of acuity [7]. For example, in three large, randomized trials of sepsis treatment, the δ-subtype was associated with greater perturbation of endothelial markers, inflammatory pathways, and coagulation dysfunction at baseline [7]. And although not definitive, many precision treatments like immune modulators or intravenous fluid choice are under investigation to uncover baseline biologic signatures and treatment response for predictive enrichment [23].

These data confirmed our hypothesis that more rapid intravenous fluid treatment, as an undifferentiated approach, or even by subtype, is not associated with improved risk-adjusted outcomes in septic shock [24]. In our model of time to completion of the weight-based intravenous fluid bolus, we did not include patients who received less than a 30 mL per kg bolus and did not assess association between total weight-based fluid volume delivered and mortality. Recent trials addressing intravenous fluid type, pace of resuscitation, and volume of fluid bolus have not resolved a beneficial approach [25–30]. In fact, a promising fluid management approach may derive from artificial intelligence powered models that adjust hemodynamic support every few hours across hundreds of patient states [31, 32]. These models are far more complex than a single-time-point-assessed subtype and need testing in randomized trials. Understanding total fluid volume, in addition to timing of fluids, may help further understand the relationship between fluids and mortality.

Our study has several limitations. First, this is an observational study and may be biased by unmeasured confounding [33]. Among many potential confounders, the appropriateness of broad-spectrum antibiotics was not measured in the NYSDOH clinical database. The appropriateness of initial antibiotic choice has been associated with risk-adjusted mortality [19], but our E-value analysis revealed that potential confounders would need substantial imbalance across groups as well as a strong independent association with outcome to obviate the main study result [14]. Available NYSDOH data was limited to 12 h after protocol initiation for completion of the 3-h bundle and antibiotic initiation and to six hours after protocol initiation for completion of the weight-based fluid intervention. Thus, we were unable to assess the potential impact of element completion beyond these time windows. Second, the results were reported from hospitals who were subject to the same state mandate for protocolized care [16]. The epidemiology of sepsis, clinical subtypes, and timing of interventions may be different in other regions [34, 35]. The cohort was limited to patients with community-acquired sepsis, which may limit the external validity of our results. Third, the identification of clinical sepsis subtypes using the SENECA algorithm involves more than 20 variables in the EHR, and not all proposed variables were present in the NYSDOH dataset. In prior work, SENECA classifications are robust to multiple data sources, feature availability, and missingness [7, 36]. Fourth, care practices may be different in a more contemporary dataset, though recent studies of the NY sepsis mandate reveal similar case mix and process measures [37].

## Conclusion

More rapid completion of the 3-h bundle and administration of antibiotics, particularly among the δ-subtype, was associated with lower risk-adjusted in-hospital mortality.

## Supplementary Information

**Additional file 1**: **Methods**. **Fig. S1**. Lowess-smoothed hospital mortality over time to completion of the 3-hour bundle, overall and by sepsis subtype. **Fig. S2**. Crude in-hospital mortality and predicted risks of in-hospital death by time to intravenous fluid bolus completion by sepsis subtype. **Table S1**. Baseline characteristics among encounters receiving intravenous fluid bolus. **Table S2**. Odds ratio of in-hospital mortality with 95% confidence interval for time to completing the 3-hour bundle overall and by sepsis subtype. **Table S3**. Odds ratio of in-hospital mortality with 95% confidence interval for time to administration of broad-spectrum antibiotics overall and by sepsis subtype **Table S4**. Odds ratio of in-hospital mortality with 95% confidence interval for time to completion of initial intravenous fluid bolus completion overall and by sepsis subtype. **Table S5**. Odds ratio of in-hospital mortality with 95% confidence interval for time to obtain initial blood cultures and time to initial lactate result, across predicted cluster assignment. **Table S6**. e-Values for logistic regression model of in-hospital mortality by time to completing the 3-hour bundle overall and by sepsis subtype. **Table S7**. e-Values for logistic regression model of in-hospital mortality by time to administration of broad-spectrum antibiotics overall and by sepsis subtype. **Table S8**. e-Values for logistic regression model of in-hospital mortality by time to completion of initial intravenous fluid bolus overall and by sepsis subtype.

## Data Availability

Data supporting the results reported in the article can be found in the Supplementary Appendix, as follows:
