# Supplementary material for: Time to treatment and mortality for clinical sepsis subtypes
Source: Crit Care. 2023 Jun 15;27:236. doi: 10.1186/s13054-023-04507-5 (PMC10268363; doi:10.1186/s13054-023-04507-5)
Supplement: Supplementary file 1 — Additional file 1: Methods. Fig. S1. Lowess-smoothed hospital mortality over time to completion of the 3-hour bundle, overall and by sepsis subtype. Fig. S2. Crude in-hospital mortality and predicted risks of in-hospital death by time to intravenous fluid bolus completion by sepsis subtype. Table S1. Baseline characteristics among encounters receiving intravenous fluid bolus. Table S2. Odds ratio of in-hospital mortality with 95% confidence interval for time to completing the 3-hour bundle overall and by sepsis subtype. Table S3. Odds ratio of in-hospital mortality with 95% confidence interval for time to administration of broad-spectrum antibiotics overall and by sepsis subtype Table S4. Odds ratio of in-hospital mortality with 95% confidence interval for time to completion of initial intravenous fluid bolus completion overall and by sepsis subtype. Table S5. Odds ratio of in-hospital mortality with 95% confidence interval for time to obtain initial blood cultures and time to initial lactate result, across predicted cluster assignment. Table S6. e-Values for logistic regression model of in-hospital mortality by time to completing the 3-hour bundle overall and by sepsis subtype. Table S7. e-Values for logistic regression model of in-hospital mortality by time to administration of broad-spectrum antibiotics overall and by sepsis subtype. Table S8. e-Values for logistic regression model of in-hospital mortality by time to completion of initial intravenous fluid bolus overall and by sepsis subtype. [file 13054_2023_4507_MOESM1_ESM.docx]

**Supplementary Appendix**

This appendix has been provided by the authors to give readers additional information about their work.

Supplement to: Yang A, Kennedy JN, Reitz KM, et al. Time to Treatment and Mortality for Sepsis Clinical Subtypes.

**Supplementary Appendix**

**Time to Treatment and Mortality for Sepsis Clinical Subtypes**

Table of Contents Page

[eMethods 2](#_Toc129529292)

[eFigure 1. Lowess-smoothed hospital mortality over time to completion of the 3-hour bundle, overall (top) and by sepsis subtype (bottom); N = 55,169 4](#_Toc129529293)

[eTable 1. Baseline characteristics among encounters receiving IV fluid bolus 6](#_Toc129529294)

[eTable 2. Odds ratio of in-hospital mortality with 95% confidence interval for time to completing the 3-hour bundle overall and by sepsis subtype 7](#_Toc129529295)

[eTable 3. Odds ratio of in-hospital mortality with 95% confidence interval for time to administration of broad-spectrum antibiotics overall and by sepsis subtype 8](#_Toc129529296)

[eTable 4. Odds ratio of in-hospital mortality with 95% confidence interval for time to completion of initial IV fluid bolus overall and by sepsis subtype 9](#_Toc129529297)

[eTable 5. Odds ratio of in-hospital mortality with 95% confidence interval for time to obtain initial blood cultures and time to initial lactate result, across predicted cluster assignment 10](#_Toc129529298)

[eTable 6. e-Values for logistic regression model of in-hospital mortality by time to completing the 3-hour bundle overall and by sepsis subtype 11](#_Toc129529299)

[eTable 7. e-Values for logistic regression model of in-hospital mortality by time to administration of broad spectrum antibiotics overall and by sepsis subtype 12](#_Toc129529300)

[eTable 8. e-Values for logistic regression model of in-hospital mortality by time to completion of initial IV fluid bolus overall and by sepsis subtype 13](#_Toc129529301)

# eMethods

Here, we provide supplemental methodology on Time to Treatment and Mortality for Sepsis Clinical Subtypes.

*Sepsis Identification Strategies Across New York State Hospitals*

New York hospitals were instructed to identify severe sepsis and septic shock cases using

protocols with criteria suggested in Sepsis-2.0.^1^ In the case report form, four categories were used to categorize how cases were identified at each facility.

1. Positive sepsis screening from clinical assessment

- This category indicates that the facility used some form of screening of ED patients or inpatients, and the result was positive for sepsis/severe sepsis/septic shock
- For many facilities, the sepsis screen tool will utilize an assessment for at least 2 of the 4 SIRS criteria (temp, heart rate, respiratory rate, WBC’s) and a suspected/confirmed infection
- This screening process can also incorporate clinical assessments for blood pressure/hypotension, altered mental status, etc.
- Laboratory values (e.g. WBCs, serum lactate levels, creatinine, bilirubin, etc.) may also contribute to a positive screening, but for this category, lab values are not always necessary to reach a positive screening result

1. Positive sepsis screen from clinical assessment AND abnormal laboratory values

- This category includes the components of the previous category plus laboratory values
- In some facilities when the initial screening of a patient comes back as positive for possible sepsis, immediate laboratory tests are ordered. The clinician then used the laboratory values in deciding whether or not to initiate their protocol
- For this category, any laboratory value can be used that supports the sepsis assessment qualifies (WBC counts, % of band cells, platelet counts, serum lactate, blood glucose, creatinine, bilirubin, C-reactive protein, procalcitonin, coagulation abnormalities-INR/aPTT, blood gases)

1. Positive sepsis screen from clinical assessment AND code sepsis

- This category includes a positive sepsis screen by clinical assessment
- However, it identifies patients in whom a code sepsis or rapid response team call initiates the case identification

*Consort Diagram Exclusions*

Clinical contraindications: Clinical contraindications include patients with administrative contraindication to care, patients enrolled in a clinical trial for sepsis, severe sepsis or septic shock treatment or intervention, patients who were discharged within 6 hours of presentation, patients receiving IV antibiotics for more than 24 hours prior to presentation, and/or patients pregnant at 20 weeks through Day 3 post-delivery.

Advanced directives: Advanced directives are defined as patients who had a directive for Comfort Care or Palliative Care.

*Imputation*

We assumed missing at random (MAR) for this study and imputed 572 missing initial serum lactates (0.99%) out of 57,771 observations, including the study population as well as patients in whom the 3-hour bundle was not completed. Imputation was performed using multiple imputation by chained equations (MICE) to create one imputed dataset. Imputed data was

appended to the original data. Odds ratios were generated using complete case data for the original data and for data with imputed lactate. Results were pooled into a single result and adjusted for the variability within and between the two datasets using Rubin’s combining rules. A random seed was specified for reproducibility of results.

Continuous serum lactate was imputed using chained imputation that specifically used a truncated linear regression where the lower limit of the imputed initial serum lactate was 0.111 mmol/L and the upper limit was 30.0 mmol/L. This regression assumed a parametric model with underlying truncated normal distribution and assigned values for missing serum lactates within this range.

The 23 variables used to impute missing serum lactate were age, sex, race, ethnicity, payer type, admission source, hospital mortality, completion of intravenous fluid bolus, hypotension, vasopressors given, platelet count, bandemia status, diagnosis of septic shock, site of infection, mechanical ventilation severity, admission to the intensive care unit, chronic respiratory failure, metastatic cancer, lymphoma, leukemia, or multiple myeloma, congestive heart failure, chronic renal failure, chronic liver disease, diabetes.

# eFigure 1. Lowess-smoothed hospital mortality over time to completion of the 3-hour bundle, overall (top) and by sepsis subtype (bottom); N = 55,169


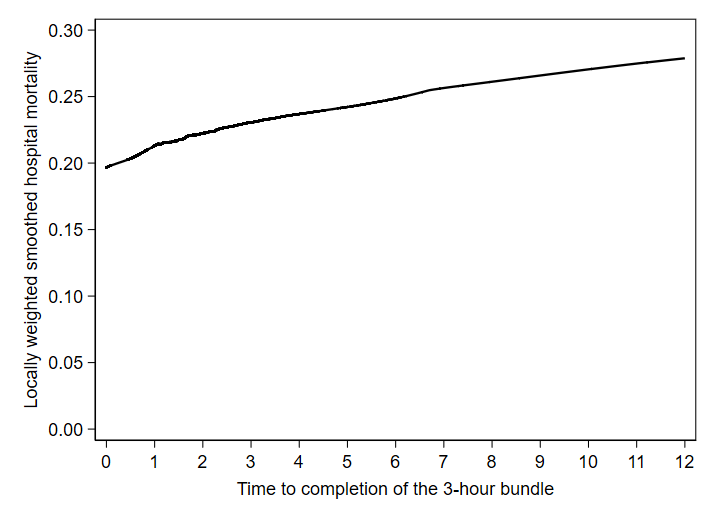


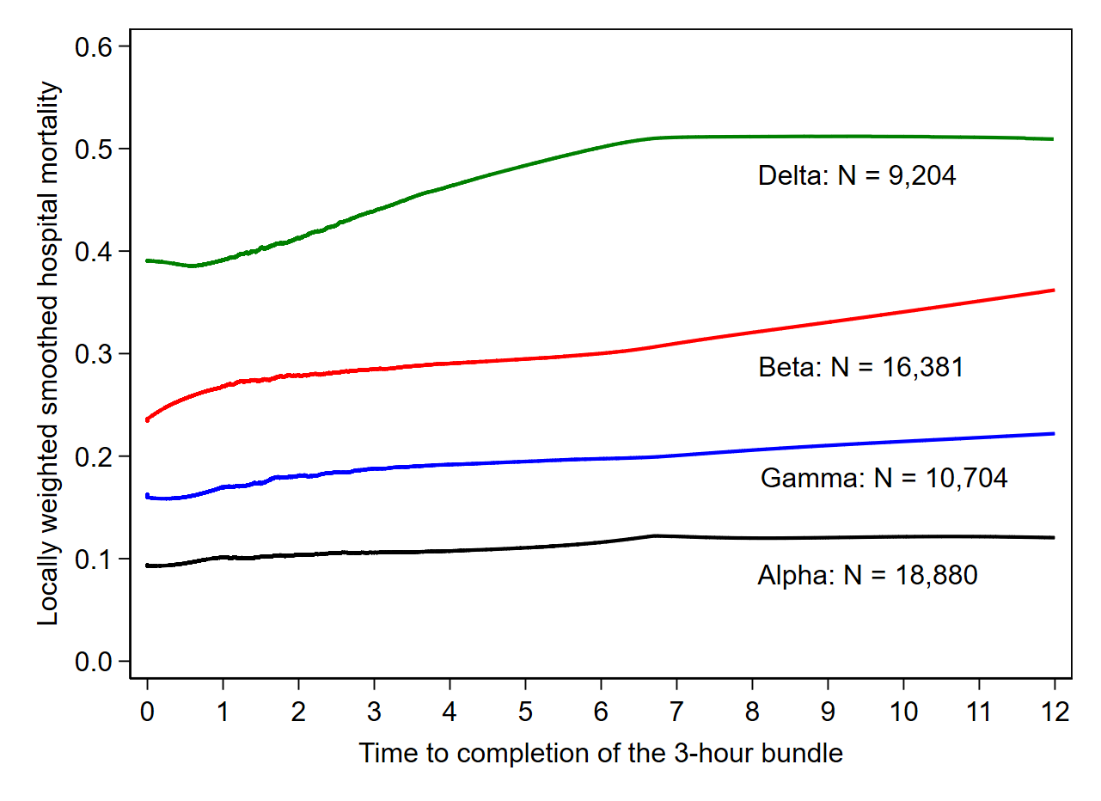


P-value for fit of linear model vs. first degree multivariable fractional polynomial

model = 0.899 for change of deviance, indicating linear fit is appropriate.

**eFigure 2.** Crude in-hospital mortality and predicted risks of in-hospital death by time to intravenous fluid bolus completion by sepsis subtype

Shown are the crude in-hospital mortality and model-estimated risks of in-hospital death for each clinical sepsis subtype, across a range of times from initiation of protocol to initial IV fluid bolus completion. Bars represent 95% confidence intervals.

# eTable 1. Baseline characteristics among encounters receiving IV fluid bolus

|  |  | **Clinical Sepsis Subtype** | | | |
| --- | --- | --- | --- | --- | --- |
|  | **All** | **⍺** | **β** | **γ** | **δ** |
| No. (%) | 26,766 | 8,558 (32) | 7,101 (27) | 5,269 (20) | 5,838 (22) |
| **Patient Demographics** |  |  |  |  |  |
| Age at admission, med. [IQR] | 71 [59-83] | 61 [49-73] | 80 [70-87] | 72 [61-83] | 72 [60-83] |
| Sex, No. (%) |  |  |  |  |  |
| Male | 13,672 (51) | 4,621 (54) | 3,365 (47) | 2,223 (42) | 3,463 (59) |
| Female | 13,094 (49) | 3,937 (46) | 3,736 (53) | 3,046 (58) | 2,375 (41) |
| Race, No. (%) |  |  |  |  |  |
| White | 17,498 (65) | 5,260 (61) | 4,897 (69) | 3,648 (69) | 3,693 (63) |
| Black | 4,432 (17) | 1,617 (19) | 1,049 (15) | 712 (14) | 1,054 (18) |
| Asian | 1,252 (4.7) | 365 (4.3) | 318 (4.5) | 270 (5.1) | 299 (5.1) |
| Other | 3,584 (13) | 1,316 (15) | 837 (12) | 639 (12) | 792 (14) |
| Admission source, No. (%) |  |  |  |  |  |
| Home | 18,331 (69) | 6,322 (74) | 4,394 (62) | 3,677 (70) | 3,938 (67) |
| Skilled nursing facility | 7,195 (27) | 1,824 (21) | 2,407 (34) | 1,288 (24) | 1,676 (29) |
| Other^b^ | 1,240 (4.6) | 412 (4.8) | 300 (4.2) | 304 (5.8) | 224 (3.8) |
| **Coexisting condition, No. (%)** |  |  |  |  |  |
| Chronic respiratory failure | 2,576 (9.6) | 345 (4.0) | 1,319 (19) | 306 (5.8) | 606 (10) |
| Congestive heart failure | 4,255 (16) | 508 (5.9) | 2,395 (34) | 516 (9.8) | 836 (14) |
| End-stage renal disease | 2,106 (7.9) | 273 (3.2) | 1,317 (16) | 232 (4.4) | 464 (7.9) |
| **Sepsis Characteristics** |  |  |  |  |  |
| Site of infection, No. (%) |  |  |  |  |  |
| Urinary | 7,601 (28) | 2,628 (31) | 1,942 (27) | 1,578 (30) | 1,453 (25) |
| Respiratory | 10,235 (38) | 3,038 (36) | 3,078 (43) | 1,871 (36) | 2,248 (39) |
| Gastrointestinal | 3,117 (12) | 766 (9.0) | 721 (10) | 788 (15) | 842 (14) |
| Other^c^ | 5,813 (22) | 2,126 (25) | 1,360 (19) | 1,032 (20) | 1,295 (22) |
| Positive blood cultures, No. (%) | 4,388 (16) | 1,082 (13) | 929 (13) | 1,038 (20) | 1,339 (23) |
| Lactate, mmol/L, med. [IQR] | 3.0 [1.9-4.9] | 2.3 [1.5-3.3] | 2.4 [1.6-3.5] | 3.1 [2.1-4.4] | 72 [5.3-9.8] |
| Septic shock^d^, No. (%) | 13,888 (52) | 2,416 (28) | 4,739 (67) | 2,545 (48) | 4,188 (72) |
| Hypotension status, No. (%) |  |  |  |  |  |
| Fluid unresponsive | 9,602 (36) | 1,899 (22) | 3,138 (44) | 1,724 (33) | 2,841 (49) |
| Responsive to fluids | 10,356 (39) | 3,333 (39) | 2,483 (35) | 2,228 (42) | 2,312 (40) |
| **In-hospital Outcomes** |  |  |  |  |  |
| Mortality, No. (%) | 6,109 (23) | 863 (10) | 1,961 (28) | 934 (18) | 2,351 (40) |
| *Abbreviations:* IQR, interquartile range; med, median; LOS, length of stay.  ^a^ Corresponds to minimum or maximum values within 6 hours of hospital admission. Comparisons were performed using the Kruskal-Wallis test or the Pearson chi-square (p < .01 for all comparisons)  ^b^ Other locations include clinic or unknown  ^c^ Other sources include skin, central nervous system, and unknown  ^d^ As defined by Sepsis-2 guidelines | | | | | |

# eTable 2. Odds ratio of in-hospital mortality with 95% confidence interval for time to completing the 3-hour bundle overall and by sepsis subtype

| **Condition** | **N** | **Mortality odds ratio^a^** | **95% CI** | **p-value^b^** |
| --- | --- | --- | --- | --- |
| *Overall* |  |  |  |  |
| Time to bundle completion, per hour | 55,169 | 1.04 | 1.02 – 1.05 | < 0.001 |
|  |  |  |  |  |
| *By Subtype* |  |  |  |  |
| Time to bundle completion by subtype, per hour |  |  |  |  |
| · Alpha | 18,880 | 1.02 | 0.99 – 1.04 | 0.139 |
| · Beta | 16,381 | 1.04 | 1.02 – 1.06 | < 0.001 |
| · Gamma | 10,704 | 1.04 | 1.01 – 1.07 | 0.003 |
| · Delta | 9,204 | 1.07 | 1.05 – 1.10 | < 0.001 |
| ^a^ Mortality odds ratio per 1 hour increase in time to completion of the 3-hour bundle | | | | |
| ^b^ Omnibus p-value for interaction = 0.019 | | | | |
|  |  |  |  |  |
| Random-effects logistic regression models with hospital as random effect and in-hospital mortality as outcome. The overall model has continuous time to completion of the 3-hour bundle as primary risk factor; the by subtype model has the interaction of subtype and continuous time to completion of the 3-hour bundle as primary risk factor, both within 12 hours of hospital arrival. | | | | |

# eTable 3. Odds ratio of in-hospital mortality with 95% confidence interval for time to administration of broad-spectrum antibiotics overall and by sepsis subtype

| **Condition** | **N** | **Mortality odds ratio^a^** | **95% CI** | **p-value^b^** |
| --- | --- | --- | --- | --- |
| *Overall* |  |  |  |  |
| Time to start of antibiotics, per hour | 55,169 | 1.03 | 1.02 – 1.04 | < 0.001 |
|  |  |  |  |  |
| *By Subtype* |  |  |  |  |
| Time to start of antibiotics by subtype, per hour |  |  |  |  |
| · Alpha | 18,880 | 1.03 | 1.00 – 1.05 | 0.081 |
| · Beta | 16,381 | 1.04 | 1.02 – 1.06 | < 0.001 |
| · Gamma | 10,704 | 1.02 | 0.99 – 1.05 | 0.175 |
| · Delta | 9,204 | 1.07 | 1.05 – 1.10 | < 0.001 |
| ^a^ Mortality odds ratio per 1 hour increase in time to start of antibiotics | | | | |
| ^b^ Omnibus p-value for interaction = 0.045 | | | | |
|  |  |  |  |  |
| Random-effects logistic regression models with hospital as random effect and in-hospital mortality as outcome. The overall model has continuous time to start of antibiotics as primary risk factor; the by subtype model has the interaction of subtype and continuous time to start of antibiotics as primary risk factor, both within 12 hours of hospital arrival. | | | | |

# eTable 4. Odds ratio of in-hospital mortality with 95% confidence interval for time to completion of initial IV fluid bolus overall and by sepsis subtype

| **Condition** | **N** | **Mortality odds ratio^a^** | **95% CI** | **p-value^b^** |
| --- | --- | --- | --- | --- |
| *Overall* |  |  |  |  |
| Time to fluid bolus completion, per hour | 26,766 | 0.99 | 0.97 – 1.01 | 0.35 |
|  |  |  |  |  |
| *By Subtype* |  |  |  |  |
| Time to fluid bolus completion by subtype, per hour | |  |  |  |
| · Alpha | 8,558 | 0.96 | 0.92 – 1.00 | 0.072 |
| · Beta | 7,101 | 1.00 | 0.97 – 1.04 | 0.889 |
| · Gamma | 5,269 | 1.00 | 0.96 – 1.05 | 0.904 |
| · Delta | 5,838 | 1.00 | 0.96 – 1.03 | 0.912 |
| ^a^ Mortality odds ratio per 1 hour increase in time to completion of IV fluid administration | | | | |
| ^b^ Omnibus p-value for interaction = 0.407 | | | | |
|  |  |  |  |  |
| Random-effects logistic regression models with hospital as random effect and in-hospital mortality as outcome. The overall model has continuous time to completion of a 30 ml bolus of intravenous fluids per kilogram of body weight; the by subtype model has the interaction of subtype and time to fluid bolus completion as primary risk factor, both within 6 hours of hospital arrival. | | | | |

# eTable 5. Odds ratio of in-hospital mortality with 95% confidence interval for time to obtain initial blood cultures and time to initial lactate result, across predicted cluster assignment

| **Bundle Element^a^** | **All patients** | **Clinical Sepsis Subtype** | | | | **Omnibus *p*-value** |
| --- | --- | --- | --- | --- | --- | --- |
|  |  | **Alpha** | **Beta** | **Gamma** | **Delta** |  |
| Time to when blood cultures  are obtained ≤ 12 hours | 1.05  (1.03 – 1.06)  *p*: < 0.001 | 1.01  (0.97 – 1.04) | 1.05  (1.02 – 1.07) | 1.04  (1.01 – 1.08) | 1.07  (1.04 – 1.11) | 0.064 |
| Time to when initial lactate  was reported ≤ 12 hours | 1.05  (1.03 – 1.07)  *p*: < 0.001 | 1.03  (0.99 – 1.07) | 1.06  (1.03 – 1.09) | 1.06  (1.02 – 1.10) | 1.07  (1.03 – 1.12) | 0.518 |

^a^ Mortality odds ratio for 1 hour increase in either time to obtain first blood cultures or report of initial lactate

# eTable 6. e-Values for logistic regression model of in-hospital mortality by time to completing the 3-hour bundle overall and by sepsis subtype

| **Condition** | **Mortality odds ratio** | **95% CI** | **E-value and lower confidence limit** |
| --- | --- | --- | --- |
| *Overall* |  |  |  |
| Time to bundle completion, per hour | 1.04 | 1.02 – 1.05 | 1.16 and 1.11 |
|  |  |  |  |
| *By Subtype* |  |  |  |
| Time to bundle completion by subtype, per hour |  |  |  |
| · Alpha | 1.02 | 0.99 – 1.04 | 1.11 and 1.00 |
| · Beta | 1.04 | 1.02 – 1.06 | 1.16 and 1.11 |
| · Gamma | 1.04 | 1.01 – 1.07 | 1.16 and 1.08 |
| · Delta | 1.07 | 1.05 – 1.10 | 1.22 and 1.18 |
| E-value represents the minimum strength of unmeasured confounder(s) necessary to nullify the main effect. E-Value to nullify the overall effect modification of mortality odds ratio by clinical sepsis subtype is 1.18 (Lower confidence limit: 1.11). | | | |

# eTable 7. e-Values for logistic regression model of in-hospital mortality by time to administration of broad spectrum antibiotics overall and by sepsis subtype

| **Condition** | **Mortality odds ratio** | **95% CI** | **E-value and lower confidence limit** |
| --- | --- | --- | --- |
| *Overall* |  |  |  |
| Time to bundle completion, per hour | 1.03 | 1.02 – 1.04 | 1.14 and 1.11 |
|  |  |  |  |
| *By Subtype* |  |  |  |
| Time to bundle completion by subtype, per hour |  |  |  |
| · Alpha | 1.03 | 1.00 – 1.05 | 1.14 and 1.00 |
| · Beta | 1.04 | 1.02 – 1.06 | 1.16 and 1.11 |
| · Gamma | 1.02 | 0.99 – 1.05 | 1.11 and 1.00 |
| · Delta | 1.07 | 1.05 – 1.10 | 1.22 and 1.18 |

E-value represents the minimum strength of unmeasured confounder(s) necessary to nullify the main effect. E-Value to nullify the overall effect modification of mortality odds ratio by clinical sepsis subtype is 1.16 (Lower confidence limit: 1.08).

# eTable 8. e-Values for logistic regression model of in-hospital mortality by time to completion of initial IV fluid bolus overall and by sepsis subtype

| **Condition** | **Mortality odds ratio** | **95% CI** | **E-value and lower confidence limit** |
| --- | --- | --- | --- |
| *Overall* |  |  |  |
| Time to bundle completion, per hour | 0.99 | 0.97 – 1.01 | 1.08 and 1.00 |
|  |  |  |  |
| *By Subtype* |  |  |  |
| Time to bundle completion by subtype, per hour |  |  |  |
| · Alpha | 0.96 | 0.92 – 1.00 | 1.17 and 1.00 |
| · Beta | 1.00 | 0.97 – 1.04 | 1.00 and 1.00 |
| · Gamma | 1.00 | 0.96 – 1.05 | 1.00 and 1.00 |
| · Delta | 1.00 | 0.96 – 1.03 | 1.00 and 1.00 |
| E-value represents the minimum strength of unmeasured confounder(s) necessary to nullify the main effect. E-Value to nullify the overall effect modification of mortality odds ratio by clinical sepsis subtype is 1.00 (Lower confidence limit: 1.00). | | | |

**References:**

1. [Levy MM, Fink MP, Marshall JC, et al. 2001 SCCM/ESICM/ACCP/ATS/SIS international sepsis definitions conference. *Intensive Care Med*. 2003;29(4):530-538. doi:10.1007/s00134-003-1662-x](https://sciwheel.com/work/bibliography/1631636)
